# *N*-(4-Hy­droxy­phen­yl)-4-nitro­benzamide

**DOI:** 10.1107/S1600536813006132

**Published:** 2013-03-09

**Authors:** Ghulam Waris, Humaira Masood Siddiqi, Ulrich Flörke, Shaukat Saeed, M. Saeed Butt

**Affiliations:** a Department of Chemistry, Quaid-I-Azam University, Islamabad 45320, Pakistan; b Universität Paderborn, Warburgerstrasse 100, D-33098 Paderborn, Germany; c Department of Materials and Metallurgical Engineering (DMME), Pakistan Institute of Engineering and Applied Sciences (PIEAS), Islamabad 45650, Pakistan

## Abstract

The mol­ecular structure of the title compound, C_13_H_10_N_2_O_4_, shows an almost planar conformation as the benzene rings make a dihedral angle of 2.31 (7)°. The nitro group lies in plane with the benzamide ring, with a C—C—N—O torsion angle of 0.6 (2)°. In the crystal, N—H⋯O and O—H⋯O hydrogen bonds link mol­ecules into sheets stacked along [10-1].

## Related literature

For background to aromatic polyimides, see: Sheng *et al.* (2009 ▶). For the solubilizing role of ether and amide groups in polyimides, see: Litvinov *et al.* (2010 ▶). For a related structure, see: Raza *et al.* (2010 ▶).
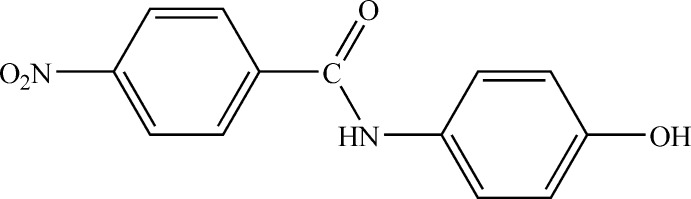


## Experimental

### 

#### Crystal data

C_13_H_10_N_2_O_4_
*M* *_r_* = 258.23
Monoclinic, 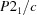

*a* = 7.5187 (5) Å
*b* = 12.5695 (9) Å
*c* = 11.7932 (8) Å
β = 90.033 (2)°
*V* = 1114.53 (13) Å^3^
*Z* = 4
Mo *K*α radiation
μ = 0.12 mm^−1^
*T* = 130 K
0.50 × 0.16 × 0.12 mm

#### Data collection

Bruker SMART APEX diffractometer
Absorption correction: multi-scan (*SADABS*; Sheldrick, 2004 ▶) *T*_min_ = 0.944, *T*_max_ = 0.986
10323 measured reflections
2657 independent reflections
2255 reflections with *I* > 2σ(*I*)
*R*_int_ = 0.030

#### Refinement

*R*[*F* ^2^ > 2σ(*F* ^2^)] = 0.048
*wR*(*F* ^2^) = 0.122
*S* = 1.12
2657 reflections
174 parameters
H-atom parameters constrained
Δρ_max_ = 0.37 e Å^−3^
Δρ_min_ = −0.28 e Å^−3^

### 

Data collection: *SMART* (Bruker, 2002 ▶); cell refinement: *SAINT* (Bruker, 2002 ▶); data reduction: *SAINT*; program(s) used to solve structure: *SHELXTL* (Sheldrick, 2008 ▶); program(s) used to refine structure: *SHELXTL*; molecular graphics: *SHELXTL*; software used to prepare material for publication: *SHELXTL* and local programs.

## Supplementary Material

Crystal structure: contains datablock(s) I, global. DOI: 10.1107/S1600536813006132/tk5203sup1.cif

Structure factors: contains datablock(s) I. DOI: 10.1107/S1600536813006132/tk5203Isup2.hkl

Supplementary material file. DOI: 10.1107/S1600536813006132/tk5203Isup3.cml

Additional supplementary materials:  crystallographic information; 3D view; checkCIF report

## Figures and Tables

**Table 1:** Hydrogen-bond geometry (Å, °)

*D*—H⋯*A*	*D*—H	H⋯*A*	*D*⋯*A*	*D*—H⋯*A*
O4—H4⋯O1^i^	0.84	1.94	2.7803 (17)	175
N1—H1*A*⋯O3^ii^	0.88	2.33	3.1664 (18)	159

Symmetry codes: (i) 
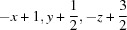
; (ii) 
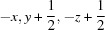
.

**Fig. 1.**
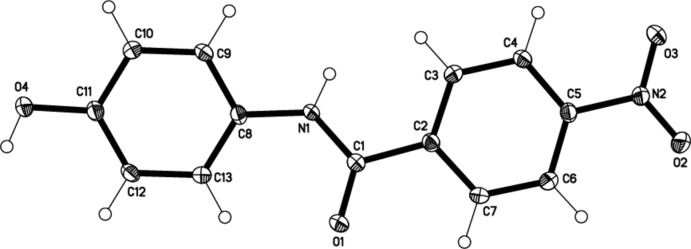
Molecular structure of the title compound with anisotropic displacement parameters drawn at the 50% probability level.

**Fig. 2.**
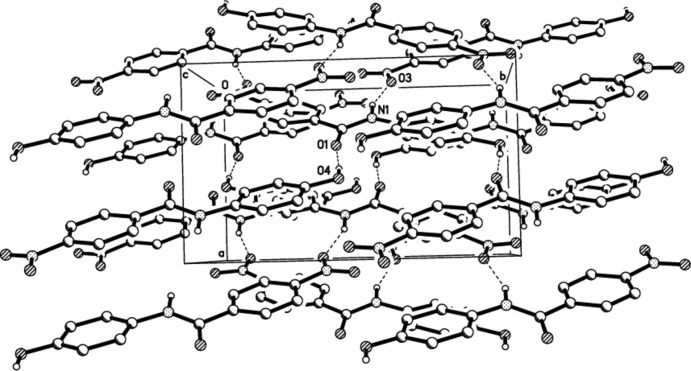
Crystal packing viewed along c axis with hydrogen bonds as dotted lines. H-atoms not involved are omitted.

## supplementary crystallographic information

### Comment

Fairly high thermooxidative and outstanding thermal stability, exceptional mechanical and electrical properties and upright chemical resistance are some distinctions of aromatic polyimides that make them documented as high performance polymeric materials (Sheng *et al.*, 2009). The reported title compound, (I), containing ether and amide groups, serve multiple purposes, such as a boost in solubility and upsurge thermal stability of the resulting polyimide (Litvinov *et al.*, 2010). In this connection, the title compound was investigated.

The molecular structure of (I), Fig. 1, is approximately planar with the dihedral angle between the two benzene rings being 2.31 (7)°. Intermolecular N—H···O and O—H···O hydrogen bonds, Table 1, link molecules into sheets stacked approximately along [1 0 1], Fig. 2. The structure of the isomeric 2-hydroxy-*N*-(3-nitrophenyl)benzamide compound is known (Raza *et al.*, 2010).

### Experimental

Reagent grade quality chemicals were used in this preparation. 4-Aminophenol (0.94 g, 8.6 mmol) in dry dichloromethane (30 ml), a few drops of *N*, *N*-dimethylformamide (DMF) and triethylamine (1.25 ml, 8.6 mmol) were placed in a 100 ml, three necked, round bottomed flask, equipped with a condenser, a nitrogen gas inlet tube, a thermometer and a magnetic stirrer. The mixture was stirred at 273–278 K for 30–45 minutes. A solution of 4-nitrobenzoyl chloride (1.59 g, 8.6 mmol) in dichloromethane (20 ml) was added drop-wise *via* a dropping funnel along with continuous stirring. The reaction mixture was then refluxed for 45 minutes. The flask contents were cooled to room temperature, poured into water and let to stand for 24 h. The resulting bright-yellow precipitate was filtered, washed with hot water and 5% NaOH solution. Finally, product was washed with hot water and dried under vacuum at 350 K. The crude product was recrystallized from ethanol and dichloromethane (2:1, *v*/*v*). Yield: 91%; m.p. 406–407 K.

### Refinement

Hydrogen atoms were clearly identified in difference syntheses, refined at idealized positions riding on the parent atoms with C—H 0.95, N—H 0.88 and O—H 0.84 Å, and with *U*_ĩso_(H) = 1.2*U*_eq_(C,N,O)

### Crystal data

**Table:**

C_13_H_10_N_2_O_4_	*F*(000) = 536
*M**_r_* = 258.23	*D*_x_ = 1.539 Mg m^−^^3^
Monoclinic, *P*2_1_/*c*	Mo *K*α radiation, λ = 0.71073 Å
Hall symbol: -P 2ybc	Cell parameters from 2515 reflections
*a* = 7.5187 (5) Å	θ = 3.2–28.1°
*b* = 12.5695 (9) Å	µ = 0.12 mm^−^^1^
*c* = 11.7932 (8) Å	*T* = 130 K
β = 90.033 (2)°	Prism, yellow
*V* = 1114.53 (13) Å^3^	0.50 × 0.16 × 0.12 mm
*Z* = 4	

### Data collection

**Table:**

Bruker SMART APEX diffractometer	2657 independent reflections
Radiation source: sealed tube	2255 reflections with *I* > 2σ(*I*)
Graphite monochromator	*R*_int_ = 0.030
φ and ω scans	θ_max_ = 27.9°, θ_min_ = 2.4°
Absorption correction: multi-scan (*SADABS*; Sheldrick, 2004)	*h* = −9→9
*T*_min_ = 0.944, *T*_max_ = 0.986	*k* = −16→16
10323 measured reflections	*l* = −15→14

### Refinement

**Table:**

Refinement on *F*^2^	Primary atom site location: structure-invariant direct methods
Least-squares matrix: full	Secondary atom site location: difference Fourier map
*R*[*F*^2^ > 2σ(*F*^2^)] = 0.048	Hydrogen site location: difference Fourier map
*wR*(*F*^2^) = 0.122	H-atom parameters constrained
*S* = 1.12	*w* = 1/[σ^2^(*F*_o_^2^) + (0.0417*P*)^2^ + 0.8505*P*] where *P* = (*F*_o_^2^ + 2*F*_c_^2^)/3
2657 reflections	(Δ/σ)_max_ < 0.001
174 parameters	Δρ_max_ = 0.37 e Å^−^^3^
0 restraints	Δρ_min_ = −0.28 e Å^−^^3^

### Special details

**Table:**

Geometry. All e.s.d.'s (except the e.s.d. in the dihedral angle between two l.s. planes) are estimated using the full covariance matrix. The cell e.s.d.'s are taken into account individually in the estimation of e.s.d.'s in distances, angles and torsion angles; correlations between e.s.d.'s in cell parameters are only used when they are defined by crystal symmetry. An approximate (isotropic) treatment of cell e.s.d.'s is used for estimating e.s.d.'s involving l.s. planes.
Refinement. Refinement of *F*^2^ against ALL reflections. The weighted *R*-factor *wR* and goodness of fit *S* are based on *F*^2^, conventional *R*-factors *R* are based on *F*, with *F* set to zero for negative *F*^2^. The threshold expression of *F*^2^ > σ(*F*^2^) is used only for calculating *R*-factors(gt) *etc*. and is not relevant to the choice of reflections for refinement. *R*-factors based on *F*^2^ are statistically about twice as large as those based on *F*, and *R*- factors based on ALL data will be even larger.

### Fractional atomic coordinates and isotropic or equivalent isotropic displacement parameters (Å^2^)

**Table:**

	*x*	*y*	*z*	*U*_iso_*/*U*_eq_	
O1	0.39417 (17)	0.43715 (9)	0.63125 (11)	0.0247 (3)	
O2	0.0650 (2)	0.00255 (10)	0.33969 (12)	0.0320 (3)	
O3	−0.01095 (17)	0.10678 (10)	0.20187 (10)	0.0239 (3)	
O4	0.41630 (19)	0.95533 (9)	0.66718 (11)	0.0284 (3)	
H4	0.4704	0.9534	0.7295	0.043*	
N1	0.23878 (18)	0.54813 (10)	0.51520 (12)	0.0176 (3)	
H1A	0.1612	0.5484	0.4593	0.021*	
N2	0.05435 (19)	0.09059 (11)	0.29598 (12)	0.0195 (3)	
C1	0.2983 (2)	0.45176 (13)	0.54794 (13)	0.0166 (3)	
C2	0.2378 (2)	0.35909 (12)	0.47682 (14)	0.0166 (3)	
C3	0.1658 (2)	0.37062 (13)	0.36841 (14)	0.0190 (3)	
H3A	0.1579	0.4393	0.3351	0.023*	
C4	0.1057 (2)	0.28251 (13)	0.30913 (14)	0.0182 (3)	
H4A	0.0541	0.2901	0.2360	0.022*	
C5	0.1225 (2)	0.18327 (12)	0.35856 (14)	0.0176 (3)	
C6	0.1982 (2)	0.16825 (13)	0.46470 (15)	0.0197 (3)	
H6A	0.2098	0.0991	0.4962	0.024*	
C7	0.2562 (2)	0.25726 (13)	0.52325 (14)	0.0186 (3)	
H7A	0.3091	0.2491	0.5959	0.022*	
C8	0.2858 (2)	0.64971 (12)	0.55977 (14)	0.0166 (3)	
C9	0.2564 (2)	0.73771 (13)	0.49077 (14)	0.0188 (3)	
H9A	0.2055	0.7280	0.4177	0.023*	
C10	0.3001 (2)	0.83960 (13)	0.52705 (15)	0.0209 (4)	
H10A	0.2796	0.8991	0.4791	0.025*	
C11	0.3742 (2)	0.85379 (13)	0.63415 (14)	0.0193 (3)	
C12	0.4006 (2)	0.76651 (13)	0.70399 (14)	0.0190 (3)	
H12A	0.4498	0.7764	0.7774	0.023*	
C13	0.3560 (2)	0.66451 (13)	0.66782 (14)	0.0189 (3)	
H13A	0.3734	0.6053	0.7167	0.023*	

### Atomic displacement parameters (Å^2^)

**Table:**

	*U*^11^	*U*^22^	*U*^33^	*U*^12^	*U*^13^	*U*^23^
O1	0.0316 (7)	0.0198 (6)	0.0226 (6)	0.0025 (5)	−0.0140 (5)	−0.0023 (5)
O2	0.0488 (9)	0.0149 (6)	0.0323 (8)	−0.0020 (5)	−0.0158 (6)	0.0000 (5)
O3	0.0293 (7)	0.0230 (6)	0.0194 (6)	−0.0011 (5)	−0.0082 (5)	−0.0039 (5)
O4	0.0435 (8)	0.0158 (6)	0.0258 (7)	−0.0030 (5)	−0.0152 (6)	−0.0019 (5)
N1	0.0214 (7)	0.0149 (6)	0.0163 (7)	−0.0002 (5)	−0.0078 (5)	−0.0012 (5)
N2	0.0219 (7)	0.0166 (7)	0.0201 (7)	0.0015 (5)	−0.0043 (6)	−0.0023 (5)
C1	0.0172 (8)	0.0168 (8)	0.0157 (7)	−0.0005 (6)	−0.0017 (6)	−0.0007 (6)
C2	0.0159 (8)	0.0166 (8)	0.0173 (8)	0.0006 (6)	−0.0023 (6)	−0.0019 (6)
C3	0.0222 (8)	0.0154 (7)	0.0194 (8)	0.0014 (6)	−0.0039 (6)	0.0012 (6)
C4	0.0197 (8)	0.0189 (8)	0.0160 (8)	0.0011 (6)	−0.0037 (6)	−0.0017 (6)
C5	0.0181 (8)	0.0155 (7)	0.0193 (8)	0.0005 (6)	−0.0025 (6)	−0.0035 (6)
C6	0.0229 (8)	0.0157 (8)	0.0204 (8)	0.0007 (6)	−0.0038 (6)	0.0018 (6)
C7	0.0215 (8)	0.0189 (8)	0.0153 (8)	0.0011 (6)	−0.0049 (6)	0.0012 (6)
C8	0.0166 (8)	0.0153 (7)	0.0180 (8)	−0.0002 (6)	−0.0018 (6)	−0.0038 (6)
C9	0.0215 (8)	0.0194 (8)	0.0154 (8)	0.0000 (6)	−0.0053 (6)	−0.0014 (6)
C10	0.0265 (9)	0.0158 (8)	0.0203 (8)	0.0001 (6)	−0.0061 (7)	0.0017 (6)
C11	0.0209 (8)	0.0164 (8)	0.0206 (8)	−0.0003 (6)	−0.0040 (6)	−0.0031 (6)
C12	0.0208 (8)	0.0204 (8)	0.0159 (8)	−0.0001 (6)	−0.0051 (6)	−0.0031 (6)
C13	0.0227 (8)	0.0175 (8)	0.0167 (8)	0.0004 (6)	−0.0042 (6)	0.0015 (6)

### Geometric parameters (Å, º)

**Table:**

O1—C1	1.232 (2)	C4—H4A	0.9500
O2—N2	1.2234 (19)	C5—C6	1.388 (2)
O3—N2	1.2303 (18)	C6—C7	1.385 (2)
O4—C11	1.3715 (19)	C6—H6A	0.9500
O4—H4	0.8400	C7—H7A	0.9500
N1—C1	1.348 (2)	C8—C9	1.391 (2)
N1—C8	1.4252 (19)	C8—C13	1.391 (2)
N1—H1A	0.8800	C9—C10	1.390 (2)
N2—C5	1.471 (2)	C9—H9A	0.9500
C1—C2	1.505 (2)	C10—C11	1.392 (2)
C2—C3	1.396 (2)	C10—H10A	0.9500
C2—C7	1.399 (2)	C11—C12	1.386 (2)
C3—C4	1.385 (2)	C12—C13	1.392 (2)
C3—H3A	0.9500	C12—H12A	0.9500
C4—C5	1.383 (2)	C13—H13A	0.9500
			
C11—O4—H4	109.5	C7—C6—H6A	121.0
C1—N1—C8	128.11 (13)	C5—C6—H6A	121.0
C1—N1—H1A	115.9	C6—C7—C2	120.87 (15)
C8—N1—H1A	115.9	C6—C7—H7A	119.6
O2—N2—O3	123.76 (14)	C2—C7—H7A	119.6
O2—N2—C5	118.84 (14)	C9—C8—C13	119.32 (15)
O3—N2—C5	117.40 (14)	C9—C8—N1	117.21 (14)
O1—C1—N1	123.81 (15)	C13—C8—N1	123.47 (14)
O1—C1—C2	120.35 (14)	C10—C9—C8	121.01 (15)
N1—C1—C2	115.83 (14)	C10—C9—H9A	119.5
C3—C2—C7	119.45 (14)	C8—C9—H9A	119.5
C3—C2—C1	123.16 (14)	C9—C10—C11	119.47 (15)
C7—C2—C1	117.40 (14)	C9—C10—H10A	120.3
C4—C3—C2	120.36 (15)	C11—C10—H10A	120.3
C4—C3—H3A	119.8	O4—C11—C12	122.33 (15)
C2—C3—H3A	119.8	O4—C11—C10	117.98 (14)
C5—C4—C3	118.63 (15)	C12—C11—C10	119.68 (15)
C5—C4—H4A	120.7	C11—C12—C13	120.82 (15)
C3—C4—H4A	120.7	C11—C12—H12A	119.6
C4—C5—C6	122.69 (15)	C13—C12—H12A	119.6
C4—C5—N2	118.13 (14)	C8—C13—C12	119.67 (15)
C6—C5—N2	119.17 (14)	C8—C13—H13A	120.2
C7—C6—C5	117.95 (15)	C12—C13—H13A	120.2
			
C8—N1—C1—O1	6.9 (3)	N2—C5—C6—C7	−178.04 (15)
C8—N1—C1—C2	−174.10 (15)	C5—C6—C7—C2	0.4 (3)
O1—C1—C2—C3	−164.50 (16)	C3—C2—C7—C6	−2.3 (3)
N1—C1—C2—C3	16.5 (2)	C1—C2—C7—C6	177.81 (15)
O1—C1—C2—C7	15.4 (2)	C1—N1—C8—C9	158.93 (17)
N1—C1—C2—C7	−163.60 (15)	C1—N1—C8—C13	−22.1 (3)
C7—C2—C3—C4	2.8 (2)	C13—C8—C9—C10	1.7 (3)
C1—C2—C3—C4	−177.28 (15)	N1—C8—C9—C10	−179.33 (16)
C2—C3—C4—C5	−1.4 (2)	C8—C9—C10—C11	−0.1 (3)
C3—C4—C5—C6	−0.5 (3)	C9—C10—C11—O4	179.79 (16)
C3—C4—C5—N2	178.57 (15)	C9—C10—C11—C12	−1.1 (3)
O2—N2—C5—C4	−178.54 (16)	O4—C11—C12—C13	179.87 (16)
O3—N2—C5—C4	1.3 (2)	C10—C11—C12—C13	0.8 (3)
O2—N2—C5—C6	0.6 (2)	C9—C8—C13—C12	−1.9 (3)
O3—N2—C5—C6	−179.59 (15)	N1—C8—C13—C12	179.11 (15)
C4—C5—C6—C7	1.0 (3)	C11—C12—C13—C8	0.7 (3)

### Hydrogen-bond geometry (Å, º)

**Table:**

*D*—H···*A*	*D*—H	H···*A*	*D*···*A*	*D*—H···*A*
O4—H4···O1^i^	0.84	1.94	2.7803 (17)	175
N1—H1*A*···O3^ii^	0.88	2.33	3.1664 (18)	159

Symmetry codes: (i) −*x*+1, *y*+1/2, −*z*+3/2; (ii) −*x*, *y*+1/2, −*z*+1/2.
